# The Effect of Third Molars on the Mandibular Anterior Crowding Relapse—A Systematic Review

**DOI:** 10.3390/dj11050131

**Published:** 2023-05-09

**Authors:** Ioannis Lyros, Georgios Vasoglou, Theodoros Lykogeorgos, Ioannis A. Tsolakis, Michael P. Maroulakos, Eleni Fora, Apostolos I. Tsolakis

**Affiliations:** 1 Department of Orthodontics, School of Dentistry, National and Kapodistrian University of Athens, 11527 Athens, Greece; mixalis_dent@yahoo.com (M.P.M.); apostso@otenet.gr (A.I.T.); 2 Private Practice, 17676 Kallithea, Greece; gio.vasoglou@gmail.com; 3 “Hatzikosta” General Hospital of Messolonghi, 30200 Messolonghi, Greece; theolyk@gmail.com; 4 Department of Orthodontics, School of Dentistry, Aristotle University of Thessaloniki, 54623 Thessaloniki, Greece; tsolakisioannis@gmail.com; 5 Department of Orthodontics, Case Western Reserve University, Cleveland, OH 44106, USA; 6 Department of Oral Medicine and Pathology and Hospital Dentistry, School of Dentistry, National and Kapodistrian University of Athens, 11527 Athens, Greece; foraeleni@gmail.com

**Keywords:** orthodontic treatment, third molars, relapse, secondary crowding, mandibular incisor crowding

## Abstract

The present systematic review updates the evidence on wisdom teeth contributing to lower incisor crowding following orthodontic treatment. Relevant literature was searched on online databases, namely Pubmed, Scopus, and Web of Science, up to December 2022. Eligibility criteria were formulated using the PICOS approach and PRISMA guidelines. Eligible research included original clinical studies involving patients previously being treated orthodontically with permanent dentition at the end of treatment, regardless of sex or age. The initial search yielded 605 citations. After considering eligibility criteria and removing duplicates, only 10 articles met the criteria for inclusion. The risk of bias of eligible studies was evaluated using the Cochrane Handbook for Systematic Reviews and Interventions tool. The majority were highly biased, mainly regarding allocation concealment, group similarity, and assessment blinding. The vast majority did not report statistically significant associations between the presence of third molars and crowding relapse. However, a minor effect has been suggested. Seemingly, there is no clear connection between mandibular third molars and incisor crowding after orthodontic treatment. The present review did not find adequate evidence to advocate preventative removal of the third molars for reasons of occlusal stability.

## 1. Introduction

In most cases, wisdom teeth do not participate in active orthodontic treatment. However, they have an impact in treatment planning, their fate being a matter of concern. Indeed, up to two thirds of surveyed orthodontists and oral surgeons believed that unerupted third molars can generate an anteriorly directed force component that culminates in mandibular incisor crowding [1]. However, Tufekçi et al. (2009) [2] reported that the majority of orthodontists in Sweden and the US believed that the erupting mandibular wisdom teeth rarely cause crowding despite their potential for generating an anteriorly oriented force. Nevertheless, significantly more American orthodontists may recommend prophylactic removal of third molars in comparison with their European colleagues. Surgeons were significantly more likely to recommend third molar extraction to prevent undesirable anterior crowding [3].

Niedzielska (2005) [4] claimed that in the case of insufficient space for the third molars to erupt, forces may be applied on adjacent teeth causing crowding, but in the case of adequate space, the tooth may erupt uneventfully. Sidlauskas and Trakiniene (2006) [5] did not find any statistically significant difference regarding mandibular crowding in young adults presenting with erupted, unerupted, or missing third molars, and they concluded that there is insufficient evidence to implicate these teeth for late mandibular crowding. Similarly, Karasawa et al. (2013) [6] and Mettes et al. (2005) [7] did not find convincing evidence to associate the presence of third molars with anterior mandibular crowding, or to recommend regular prophylactic extraction of asymptomatic impacted third molars in adolescents with the aim of preventing future incisor crowding.

In various reviews, the most popular conclusion has been on weak evidence to support the clear contribution of third molars in incisor crowding [8,9,10,11]. Bishara (1999) [12] did not find credible evidence to implicate third molars as a major etiologic factor in the post-treatment changes in incisor alignment. He suggested that the only relationship between these two events is that they occur at similar developmental timing, which is merely a temporal coincidence. On the contrary, Vasirand Robinson (1991) [13] found a small but statistically significant relationship of the mandibular third molar with late incisor crowding. Further, Cheng et al. (2018) [14] cautiously concluded that extracting third molars would be warranted to prevent future incisor irregularity.

Fastlicht (1970) [15] stated that the third molars do not exert any influence on incisor crowding. By contrast, Kaplan (1974) [16] presented evidence of lower incisors becoming crowded in the majority of the post-orthodontic treatment patients of his sample, but the effect was not significantly different between subjects whose mandibular third molars were erupted, impacted, or congenitally missing. Nevertheless, it has been suggested that a small statistically significant difference might have surfaced if a different statistical approach had been applied [13]. 

Ades et al. (1990) [17] found more severe incisal crowding attributed to the third molars, despite their conclusion that there was insignificant post-retention change between the group exhibiting congenitally missing wisdom teeth and other groups. Similarly, Richardson (1989) [11] did not relate third molars with incisor crowding relapse. Richardson and Mills (1990) [18] found that removing mandibular second molars alleviated anterior crowding and thought that this might be attributed to the decrease in the effect of third molar crowding. Little (1999) [19] observed that there was a statistically significant relapse of lower incisor irregularity in case of third molar presence, but when comparing the results between the groups with or without wisdom teeth, he claimed that there were no differences regarding the variables of interest. Van der Schoot et al. (1997) [20] concluded that the presence of the third molar could not be used as an excuse for the recurrence of crowding. In a randomized controlled study, Harradine et al. (1998) [21] concluded that preventive removal of third molars to reduce late incisor crowding could not be endorsed. This is in agreement with Cotrin et al. (2020) [22], who did not consider mandibular wisdom teeth affecting anterior crowding relapse.

Al-Balkhi (2004) [23] claimed that third molars were not to blame for lower incisor crowding relapse after the removal of interproximal contacts. These findings also persisted in the case of maintaining interproximal contacts. Southard et al. (1991) [24] estimated the tightness of interproximal contacts of posterior teeth mesial to the second molars following unilateral surgical extraction of wisdom teeth, and they did not find any significant contact alteration after the procedure. The above outcomes agree with the research of Okazaki (2010) [25], who also revealed that the emerging wisdom tooth did not change the overall interproximal force in the anterior mandibular region.

Hasegawa et al. (2013) [26] associated crowding with reduced arch length or third molar presence. Kahl-Nieke et al. (1995) [27] registered a small, statistically important connection with post-treatment crowding increase. Sheneman (1969) [28] reported greater post-orthodontic stability in individuals exhibiting congenitally missing wisdom teeth. Lindqvist and Thilander (1982) [29] found more favourable development in the arches after extraction of impacted mandibular third molars in orthodontically untreated patients. Richardson (1982) [30] found significantly greater mesial movement of first permanent molars and more crowding in patients with impacted wisdom teeth, but, most likely, however, the sizes of the teeth had a role in creating the crowding, and the study had a high risk of bias.

The data available to date preclude any firm conclusions either way about the relationship between third molars and anterior crowding. The present review attempts to update the contribution of wisdom teeth in mandibular anterior crowding following orthodontic treatment.

## 2. Materials and Methods

A protocol was formulated based on guidelines in the PRISMA-P statement [31], in agreement with the Cochrane Handbook for Systematic Reviews of Interventions [32] and the PRISMA statement [33]. The protocol regarding the present systematic review was registered on the Open Science Forum Database following the Prisma-P guidelines (Protocol: 10.17605/OSF.IO/9HPXB).

### 2.1. Eligibility Criteria

Eligibility criteria were determined following the PICOS (Participants, Intervention, Comparison, Outcomes, and Study design) process (Supplementary Table S1). Eligible research included clinical studies on patients previously treated orthodontically, with permanent dentition at the end of their orthodontic treatment, regardless of sex or age. Review and meta-analytic papers were not considered appropriate for the present systematic review.

### 2.2. Information Sources, Search Strategy and Study Selection

A search was directed in three databases (PubMed, Scopus, and Web of Science) with the aim to identify all relevant articles, regardless of language or date of publication. They were searched since inception up to December 2022. The first author (IL) designed a thorough search, appropriately adapted (Supplementary Table S2). Further, reference lists were studied extensively to unveil additional relevant literature. The retrieved studies were independently and twice evaluated by the first (IL) and third (TL) authors. Any doubts were collectively and carefully discussed between the authors. 

### 2.3. Study Selection

The first (IL) and third (TL) authors assessed the retrieved records independently and in duplicate. Although not blinded to author identity or study conclusions, they implemented the same method to evaluate the eligibility of each available record. Next, the authors discussed the results to resolve any doubts.

### 2.4. Data Collection

Authors IL and TL performed extraction of the data. A customized collection form was used to amass the following information from the selected studies: study details, the respective design and verification of eligibility, features of the subjects and appliances been used, the intervention itself, the duration of treatment, and the outcomes.

### 2.5. Risk of Bias in Individual Studies

To evaluate the risk of bias of the eligible studies, the Cochrane Handbook for Systematic Reviews of Interventions tool was used by IL and ΙAΤ, according to the methodology by Higgins et al. [32]. Emerging doubts were resolved after discussion with AIT.

## 3. Results

### 3.1. Study Selection

**Figure 1:**
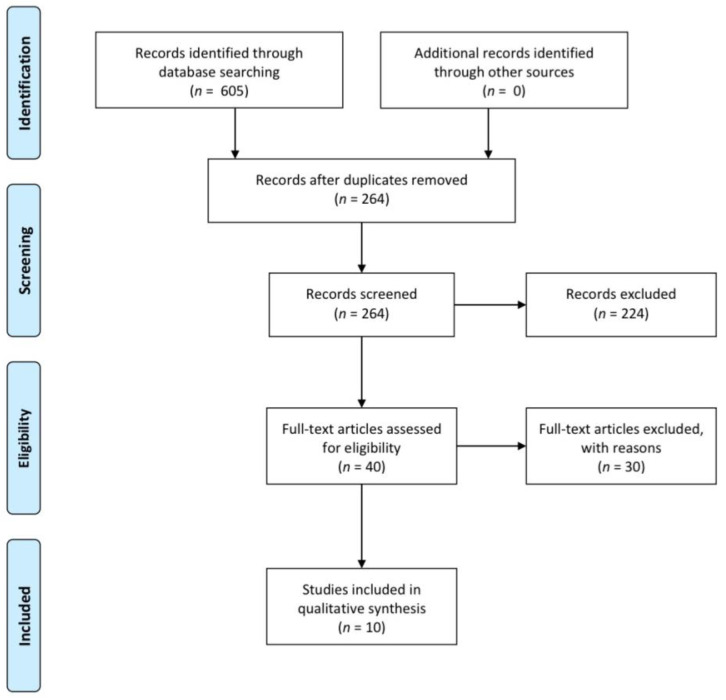
Flow chart of record selection throughout their review.

The reviewing process and the article selection are presented in Figure 1. In total, 605 records were initially retrieved. Among them, 341 proved duplicates and were excluded, as did 224 more after evaluating the titles and abstracts. For reasons such as the type of study, the outcome of interest, and the absence of previous orthodontic treatment, a further 30 records were excluded. Eventually, 10 full-text articles were deemed appropriate for inclusion in the systematic review [15,16,17,19,20,21,22,23,25,27].

### 3.2. Study Characteristics

Table 1 presents features of the included studies. The vast majority had used the Little Irregularity Index as means of measurement and plaster models for assessment.

### 3.3. Within Studies Risk of Bias 

Details regarding the risk of bias quality assessment are presented in Table 2. Eight studies were assessed for high risk of bias [15,16,17,19,20,23,25,27] and two for low risk of bias [21,22]. The majority of studies were highly biased, mainly regarding allocation concealment, group similarity, caregiver blinding, and assessment blinding.

## 4. Discussion

Human dentoalveolar structure undergoes substantial change during growth [34,35]. The process appears more pronounced until the onset of adulthood [36], but it does not cease throughout aging, and is an adaptation to environmental conditions [37,38,39,40,41]. Until the age of the late mixed dentition, the changes may become notable because facial appearance is affected by anterior tooth malalignment [42,43,44]. Consequently, anterior dental crowding is a common reason for seeking orthodontic treatment [45,46,47]. Surprisingly, though, potential relapse of tooth crowding may not prove a major disappointment for the patient, despite the orthodontist’s likely embarrassment [48,49].

In Orthodontics, relapse is defined as any unfavourable change in tooth position after orthodontic treatment that is not consistent with the corrected malocclusion [50], and the mandibular incisor imbrication is a common manifestation [19]. The eventuality of anterior crowding recurring worries orthodontists, and is inevitably observed in many treated cases [51]. In particular, it tends to happen after debonding in patients lacking retention during periodontal fiber healing [52,53,54,55]. Therefore, it is up to the orthodontist to prevent such an occurrence through planning, best practice, application of retention, and an appropriate recall regimen [56]. Nevertheless, post-treatment maintenance of mandibular arch alignment remains challenging for the clinician [57,58]. Research has tried to fully clarify the process regulating longitudinal changes in incisor arrangement, albeit with questionable credibility [8,14]. 

Angle (1907) [59] asserted that attaining occlusal stability could preserve the orthodontic therapeutic outcome [9]. This is in agreement with the finding of Kahl-Nieke et al. (1995) [27] that a perfect molar relationship may contribute to maxillary incisor alignment. However, recurrence of lower arch crowding is observed clinically quite often, even in cases with great dental intercuspation [60]. So, clinicians still attempt to preclude anterior relapse by over-correction, supra-crestal fibrotomy, and long-term retention [50,54,57]. The connection of the third molar with post-treatment relapse of malocclusion, particularly in the anterior dental arch segment, remains widely controversial and unresolved [9]. Allegedly, the erupting wisdom tooth has the potential to generate an anterior force component to be transmitted along the dental arch, concentrating in the area of the canines and incisors to result in tooth malalignment [3].

Several factors have been investigated in the search for the etiology of tooth crowding relapse, which happens after the end of active orthodontic treatment. Overall, it has been suggested that crowding in the lower anterior region and third molar impaction are both the sequela of inadequate growth [61,62,63]. In cases of restricted mandibular anterior growth and inefficient remodelling, enough space may not be generated for the mandibular incisors to move forward without getting crowded [64,65]. The effect of the tension by the neighboring soft tissues (lips, cheeks, tongue) has also been highlighted [66,67]. In addition, a mesial migration of the posterior dentition might have an effect [68] and the anteriorly oriented occlusal force component might play a role [24], while the initial orthodontic condition [27] and subsequent manipulations, (Little, 1999) the tooth dimensions [27], and the function of the periodontal tissues [57] should all not be disregarded. Lastly, various evolutionary factors [69], the gender [70,71], and the race [70,72,73] of the patient may be of importance.

Robinson (1859) [74] claimed that late lower incisor crowding is caused by the erupting mandibular permanent third molar [16]. The indictment of wisdom teeth was reaffirmed in 1917, when Dewey commented that the mandibular third molar may create space for its eruption by pushing the more anteriorly positioned teeth to move forward, potentially ending up crowded [30]. Ever since, a bulk of research has attempted to ascertain or refute the statement. Studies vary in their conclusions, several finding little relationship between third molars and late anterior tooth crowding, whereas some suggest associations of varying degree. The issue is still regarded as controversial and unresolved on the basis of emerging research, which is popular and intriguing to the dental society [14,27]. Disappointingly, Shanley (1962) [75] found insignificant differences between groups with bilaterally erupted, impacted or developmentally absent third molars, and concluded that the third molar has little influence on late anterior crowding. On the other hand, Vego (1962) [76] reported conflicting observations in a research project based on study models, without previous orthodontic treatment. He found that patients with missing wisdom teeth developed less statistically significant crowding in comparison to those with a complete dentition. So, it was concluded that erupting third molars can produce a force to approximate the teeth. However, he reported late crowding also in cases with congenitally missing wisdom teeth and, so, he suggested that mandibular malalignment might be multifactorial. It is noteworthy that Zachrisson (2005) [77] appeared to accept that lower incisor crowding during the post-orthodontic period is probably impacted by a variety of events.

The third molar is special, featuring considerable variability in the timing of formation and eruption, its course of eruption and final position, and its morphology. Erupting third molars continually change their angular positions and show important pre-eruptive rotational movement [78]. Calcification may become evident by the age of 7 years [79], commonly emerging in the mouth after the age of 17–20 years [80], competing for the highest rate of impaction and congenital absence [81,82].

In day-to-day practice, after active orthodontic treatment, the goal is to preserve the achieved therapeutic outcome. The protocol of retention should be specially planned for each patient and be carefully applied by the clinician in order to avoid unwanted occlusal relapse. In the literature, various factors have been implicated for crowding relapse, such as residual growth, sex, extractions or not, periodontal status, and dental arch expansion. The mandibular incisor crowding relapse remains unresolved and its association with mandibular third molars is still a matter of debate. Motivated by evidence-based dentistry, the current systematic review aimed to study the contribution of mandibular wisdom teeth in the re-emergence of incisor crowding.

In the present review, only Kahl-Nieke et al. (1995) [27] found a statistically significant association between wisdom teeth and lower incisor irregularity. However, the effect was considered minor and of questionable clinical value.

In 1970, Fastlicht [15] claimed that incisor crowding constitutes a normal procedure of adjustment that might be detected notwithstanding the patient’s orthodontic status. The blame was put on a number of developmental factors, namely, the sex, the age, the increased antero-posterior skull dimension, the discrepancy between dental dimension and arch length, the pronounced overbite and diminished intercanine distance, the muscular function, and even incomplete mechanotherapy. In particular, he observed that the degree of crowding increased along with mesiodistal incisor dimensions. It was also observed that crowding of the lower incisors appeared more outstanding in male patients, who measured larger maxillary and mandibular incisors in comparison with females. Overall, no connection emerged between third molars and anterior crowding in both sexes, although differences tended to be greater in women. Further, less crowding was evidenced among orthodontically treated individuals. As a result, it was suggested that orthodontic treatment might prove beneficial for occlusal stability. The age of the patients was positively related to incisor crowding, and maxillary teeth showed less irregularity.

According to Kaplan (1974) [16], most of the orthodontically treated patients may eventually end up with lower front tooth relapse. This did not correlate statistically significantly with erupted, impacted, or even congenitally missing third molars. In particular, no major changes of the dental arch length, the mandibular incisor position, or lower tooth inclination were registered throughout the period following orthodontic treatment. Furthermore, dimensional alterations of intermolar and intercanine distances happened independently of the third molar status, and they were not significant. It was of no surprise that the hypothesis of third molars applying pressure on mesial dental units could not be reasonably supported.

Ades (1990) [17] alleged that mandibular anterior crowding tends to increase by aging, while dental arch length and intercanine distance usually diminish. Post-retention records displayed only insignificant differences among the subgroups in which third molars presented congenital absence, impaction, normal eruption, or had been extracted regarding mandibular incisor crowding after considering the growth pattern. Interestingly, most investigated cases presented rather lower incisor crowding, a finding that has not been correlated to the third molars.

Van der Schoot et al. (1997) [20] claimed that the existence or absence of upper or lower third molars does not affect the long-term occurrence of the arch length variance and the anterior tooth irregularity. Indeed, when third molars were congenitally missing, the arch length discrepancy was significantly lower at the premolar area. Therefore, the presence of third molars had no noteworthy clinical connection to the developing late crowding. 

In a prospective study, Harradine et al. (1998) [21], investigated the effects of early third molar removal on late mandibular anterior crowding after the completion of retention period, and they concluded that removal of these teeth cannot be supported with robust evidence. These findings are in agreement with Linquist and Thilander (1982) [29], who investigated unilateral third molar extraction, and Vego (1962) [76], who reported on third molar agenesis. In both studies, no clinically significant effect of the wisdom teeth was found on incisor crowding, which is in agreement with the retrospective study of Ades et al. (1990) [17] involving patients who had previously been treated orthodontically. Nevertheless, these suggestions disagree with the retrospective study by Schwarze (1973) [83], concluding that wisdom tooth removal may have a benefit in alleviating later incisor crowding or improving upper arch irregularity, and also with the findings of Richardson (1996) [84] on the role of distal pressure displacing the incisors. However, there is agreement with Southard et al. (1991) [24] who believed that such a force is not significantly affected by third molar removal.

Little (1999) [19] stated that third molars’ presence or absence is not directly linked with post-retention stability or relapse, because it seemed that patients with congenital absence of mandibular third molars did not differ significantly from those having the teeth. The pilot study by Al-Balkhi (2004) [23] supported the notion that the mandibular third molars may not be major contributors in the re-appearance of incisor crowding in the case of absent intimate interproximal contacts. Nevertheless, a larger sample might be more appropriate to verify the conclusion.

Okazaki (2010) [25] alleged that increasing the total interproximal force may serve as an indication for relapse in lower incisor crowding and, so, clinicians need to be vigilant for relapse in the lower anterior segment for at least a semester following the delivery of retention when facing severe anterior incisor crowding. 

The evidence from the present systemic review cannot justify third molar extraction with the aim to prevent post-retention incisor crowding deterioration. Contrarily, extraction of the third molar may be considered in case of existing pathology, such as nerve irritation, periodontal inflammation, or increased caries risk. This is in line with the most recent past investigation by Cotrin et al. (2019) [22], who were also opposed to the recommendation for extracting the third molars to prevent potential relapse of anterior mandibular crowding because they found no connection between mandibular third molars and incisor maladjustment.

### 4.1. Strengths and Limitations

The present systematic review was constructed on established guidelines, already described in the Materials section. The reviewing process was meticulous up to December 2022, including all potentially eligible reports. 

Limitations of this systematic review might be related to the nature of the eligible articles and the features of the data. The heterogeneity of protocols in the included research and the increased risk of bias in the majority of them discouraged the realization of further meta-analysis. 

### 4.2. Recommendations for Future Research

Treatment outcome stability appears important for clinicians and patients. To achieve the goal of properly functional occlusion, patients have invested time, patience, and resources. Thus, anterior occlusal relapse constitutes an unwanted occurrence. Further randomized clinical studies, in accordance with ethical codes, are required to exclude such potentially disappointing results.

## 5. Conclusions

Considering the findings of all the above studies, we assert that there is no proven connection between mandibular wisdom teeth and lower anterior crowding relapse after orthodontic treatment. In the present systematic review, several factors likely to participate in the deterioration of tooth alignment after the actual orthodontic treatment were discussed.

## Figures and Tables

**Table 1:** Details of included studies. CG: control group, EG: experimental group, F: female, M: male, y: years, m: months, < or >or =level of statistical significance, ≈: statistically non-significant difference.

Articles	Population/Age Mean	Intervention	Compared With	Outcome of Interest of the Studies Method of Measurement	Method of Assessment	Results
Fastlicht, 1970 [15]	Total: 99, 60 F and39 M EG: 19 y 8 m; CG: 19 y 10 m	Participants with Class II, division 1 treated to normal occlusion.	EG: Orthodontic treatment (n = 28, 15 F, 13 M) CG: No orthodontic treatment (n = 28, 15 F, 13 M)	Mesiodistal incisor size, Intercanine width	Plaster models, cephalometric radiographs, photos before and after treatment	Crowding of mandibular incisors with existing 3rd molars: EG: *p* = 0.05 CG: *p* = 0.10
Kaplan., 1974 [16]	Total: 75, 48 F and 27 M	Orthodontic treatment in Class I, Class II division 1, Class II division 2 malocclusion	Orthodontically treated subjects with mandibular 3rd molars: EG1: bilaterally erupted into function, EG2: bilaterally impacted, EG3: bilateral agenesis	Arch length, Intermolar width, Intercanine width, Lower anterior crowding, Lower anterior rotations, IMPA, Lower incisor x coordinate, Lower molar x coordinate	Plaster models Cephalometric analysis	No difference between groups
Ades et al., 1990 [17]	Total: 97 28 y 6 m	Orthodontic treatment in Class I, Class II division 1, Class II division 2 malocclusion	Orthodontically treated subjects with mandibular 3rd molars: EG1: impacted EG2: erupted into function EG3: congenitally absent EG4:extracted at least 10 y earlier	Irregularity index, Mandibular intercanine width, Mandibular arch length, Overbite, Overjet, IMPA, Lower incisor x coordinate, Lower incisor y coordinate, Lower incisor angle to x axis, Lower first molar x coordinate, Lower first molar y coordinate, Lower first molar angle to axis	Plaster models, Cephalometric analysis	Mandibular anterior crowding. EG1: 3rd molars erupted: x = 3.19; SD = 2.20 EG2: Bilateral 3rd molar impaction: x = 2.27; SD = 1.81 EG3: Bilateral 3rd molar agenesis: x = 2.55; SD = 1.40 EG4: Bilateral 3rd molar extraction: x = 3.25; SD = 5.34 (f = 0.3130) Intercanine width: returned to original dimension during postretention period (x: −1.7 ± 1.4 mm; *p* ≤ 0.01); Length of arch: diminished significantly in all groups without significant Difference between groups No difference between groups
Kahl-Nieke et al., 1995 [27]	Total: 226, 131 F and 95 M Pre-treatment: mean age 11.3 y; post-treatment: mean age 15.5 y; post-retention: mean age 31.2 y	Orthodontically treated subjects	T1: Pre-treatment, T2: Post-treatment, T3: Post-retention EG1: post-retention changes ≤3 mm, EG2: post-retention changes >3 mm	Intercanine width, Intermolar width, Arch length, Little irregularity index, Crowding, Overbite, Overjet, Occlusion	Plaster models	Slightly greater irregularity index in the 3rd molar group
van der Schoot et al., 1997 [20]	Total: 99, 60 F and 39 M Pre-treatment: mean 12.8 y; post-treatment: mean 15 y; post-retention: mean 22.3 y	Orthodontically treated subjects	EG1: Right 3rd molar (mandibular arch, n = 24; maxillary arch, n = 23), EG2: No erupted 3rd molar (mandibular arch, n = 19; maxillary arch, n = 22), EG3: Right and left 3rd molars extracted (mandibular arch, n = 47; maxillary arch, n = 37), EG4: One or both 3rd molars congenitally missing (mandibular arch, n = 8; maxillary arch, n = 7)	Arch length discrepancy of the maxillary and mandibular front/anterior teeth, Arch length discrepancy of the left and right premolar area, Irregularity index of maxillary and mandibular front/anterior teeth	Plaster models, Panoramic radiographs	No significant difference between the groups (t1, t2, and t3) regarding the irregularity index in both arches. Improvement between t1–t2, deterioration between t2–t3 (*p* > 0.5)
Harradine et al., 1998 [21]	Total: 77, 45 F and 32 M 14y 10 m	Orthodontically treated subjects without use of appliances and/or retainer bar	EG1: 3rd molar removed (n = 44), EG2: 3rd molar maintained (n = 33)	Little’s index of irregularity, Intercanine width, Arch length	Plaster models, Lateral cephalometric radiographs	Value of irregularity: 3rd molar extraction, 0.80 mm 3rd molar not extracted, 1.10 mm (*p* =0.55) Intercanine width: no clinical/statistical difference Arch length: small reduction, Small statistical difference (*p* = 0.0001) in the group with no extraction (2.1 mm) in comparison with the extraction group (1.1 mm)
Little, 1999 [19]	Patients pre-treatment, post-treatment, and 10 y post-retention.		EG1: Bilateral 3rd molar impaction (n = 14) EG2: Erupted 3rd molars (n = 32) EG3: Bilateral agenesis of 3rd molars (n = 17)	Incisal irregularity index, Arch length, Intercanine width	Plaster models, Lateral cephalometric radiographs	The incisal irregularity increased in all groups; Arch length: reduced in all groups; Intercanine width: reduced in all groups
Al-Balkhi, 2004 [23]	Total: 32 14–19 y	Orthodontically treated subjects without retainer in the lower arch	EG1: Re-crowded lower incisors EG2: Uncrowded lower incisors	Crowding	Panoramic evaluation, plaster models.	EG1 ≈ EG2
Okazaki, 2010 [25]	Total: 40, 36 F and 4 M mean 23.9 y	Orthodontically treated subjects with four premolars extracted, Wrap-around retainer	T1: 0–3 m T2: 3–6 m T3: 6–12 m T4: 12–18 m	Interproximal force (IPF) evaluation in mandibular anterior region Irregularity index	Mandibular arch Plaster casts	Correlations between IPF and irregularity index: T1, T2: *p* > 0.05 T3, T4: *p* < 0.05
Cotrin et al., 2019 [22]	Total: 108	Orthodontically treated subjects	T1: Pre-treatment T2: Post-treatment T3: At least 3y post-retention EG1: 72 (39 F, 33 M) EG2: 36 (18 F, 18 M)	Little’s irregularity index	Plaster casts	EG1 ≈ EG2

**Table 2:** Summary of risk of bias assessment.

	Signalling Questions									
Study	1	2	3	4	5	6	7	8	9	Summary
Fastlicht, 1970 [15]	L	H	L	H	L	L	L	L	L	H
Kaplan., 1974 [16]	H	L	U	L	U	U	U	L	L	H
Ades et al., 1990 [17]	H	H	L	L	L	L	L	L	L	H
Kahl-Nieke et al., 1995 [27]	H	H	U	L	U	U	U	L	L	H
van der Schoot et al., 1997 [20]	H	H	U	L	U	U	L	L	L	H
Harradine et al., 1998 [21]	L	L	L	L	L	L	L	L	L	L
Little 1999 [19]	H	H	U	H	U	U	U	U	L	H
Al-Balkhi, 2004 [23]	L	L	U	H	U	U	U	L	L	H
Okazaki, 2010 [25]	L	L	H	L	H	U	L	L	L	H
Cotrin et al., 2019 [22]	U	L	U	L	U	U	L	L	L	L

(1.) Was the allocation sequence adequately generated and concealed? (2.) Were the groups similar at baseline or were they adjusted for confounders in the analysis? (3.) Were the participants, caregivers and investigators blinded? (4.) Application of inclusion and exclusion criteria for subjects.(5.)Was the outcome assessor blinded? (6.) Were incomplete outcome data adequately addressed? (7.) Are reports of the study free of selective outcome reporting? (8.) Was the statistical analysis adequate?(9.) Was the study apparently free of other bias? (H: High; L: Low; U: Unclear.)

## Data Availability

The data presented in this study are available in the included studies of this systematic review.

## Supplementary Materials

The following supporting information can be downloaded at: https://www.mdpi.com/article/10.3390/dj11050131/s1, Table S1: Eligibility criteria for the present systematic review; Table S2: Strategy for database search (up to December 2022).
