# Supplementary material for: The Effect of Third Molars on the Mandibular Anterior Crowding Relapse—A Systematic Review
Source: Dent J (Basel). 2023 May 9;11(5):131. doi: 10.3390/dj11050131 (PMC10217727; doi:10.3390/dj11050131)
Supplement: Supplementary file 1 [file dentistry-11-00131-s001.zip › Supplementary material.pdf]

Supplementary Table S1. Eligibility criteria for the present systematic review.

Domain	Inclusion criteria	Exclusion criteria
**Participants**	▪ Patients previously treated orthodontically with permanent dentition at the end of treatment.	▪ Patients previously not been treated orthodontically
**Interventions**	▪ Present, absent, extracted or congenitally missing third molars	
**Comparisons**	▪ Intervention or no intervention	
**Outcomes**	▪ Quantitative macroscopic data regarding mandibular incisor crowding [i.e. Little irregularity index] measured mainly on casts	▪ Quantitative macroscopic data regarding mandibular incisor crowding not been associated with third molars
**Studydesign**	▪ Experimental controlled studies (according to the Scottish Intercollegiate Guidelines Network algorithm for classifying study design (available at http://www.sign.ac.uk/ assets/study_design.pdf)	▪ Reviews, systematic reviews and meta-analyses

Supplementary Table S2. Strategy for database search (up to October 2020).

Database	Search strategy	Hits
**General Sources**		
**PubMed**<br>http://www.ncbi.nlm.nih.gov/pubmed	("third molar" OR "third molars" OR "wisdom tooth" OR "wisdom teeth" OR "third molar impaction" OR "third molar eruption" OR "third molar extraction" OR "third molar preventive extraction" OR "missing third molar" OR "missing wisdom tooth") AND ("crowding" OR "incisor crowding" OR "mandibular incisor crowding" OR "late lower incisor crowding" OR "secondary crowding" OR "relapse" OR "orthodontic relapse")	**188**
**Scopus**<br>https://www.scopus.com/search/form.url?zone=TopNavBar&origin=searchbasic	TITLE-ABS-KEY:<br>("third molar" OR "third molars" OR "wisdom tooth" OR "wisdom teeth" OR "third molar impaction" OR "third molar eruption" OR "third molar extraction" OR "third molar preventive extraction" OR "missing third molar" OR "missing wisdom tooth") AND ("crowding" OR "incisor crowding" OR "mandibular incisor crowding" OR "late lower incisor crowding" OR "secondary crowding" OR "relapse" OR "orthodontic relapse")	**203**
**Web of Science™**<br>http://apps.webofknowledge.com/	TITLE:<br>("third molar" OR "third molars" OR "wisdom tooth" OR "wisdom teeth" OR "third molar impaction" OR "third molar eruption" OR "third molar extraction" OR "third molar preventive extraction" OR "missing third molar" OR "missing wisdom tooth") AND ("crowding" OR "incisor crowding" OR "mandibular incisor crowding" OR "late lower incisor crowding" OR "secondary crowding" OR "relapse" OR "orthodontic relapse")<br>Timespan: All years; Search language=Auto	**214**
